# Correlation of Intravascular Ultrasound with Histology in Pediatric Pulmonary Vein Stenosis

**DOI:** 10.3390/children8030193

**Published:** 2021-03-04

**Authors:** Ryan Callahan, Zachary Gauthier, Shuhei Toba, Stephen P. Sanders, Diego Porras, Sara O. Vargas

**Affiliations:** 1 Department of Cardiology, Boston Children’s Hospital and Harvard Medical School, Boston, MA 02115, USA; Zachary.Gauthier@cardio.chboston.org (Z.G.); toba.shuhei@gmail.com (S.T.); Stephen.Sanders@cardio.chboston.org (S.P.S.); Diego.Porras@CARDIO.CHBOSTON.ORG (D.P.); 2 Department of Pathology, Boston Children’s Hospital and Harvard Medical School, Boston, MA 02115, USA; Sara.Vargas@childrens.harvard.edu

**Keywords:** pulmonary vein stenosis, intravascular ultrasound, congenital heart disease, catheterization

## Abstract

Preliminary intravascular ultrasound (IVUS) images of suspected pediatric intraluminal pulmonary vein stenosis (PVS) demonstrate wall thickening. It is unclear how the IVUS-delineated constituents of wall thickening correlate with the histology. We analyzed six postmortem formalin-fixed heart/lung specimens and four live patients with PVS as well as control pulmonary veins using IVUS and light microscopic examination. In PVS veins, IVUS demonstrated wall thickening with up to two layers of variable echogenicity, often with indistinct borders. Histologically, the veins showed fibroblastic proliferation with areas rich in myxoid matrix as well as areas with abundant collagen and elastic fibers. Discrete vein layers were obscured by scarring and elastic degeneration. A lower reflective periluminal layer by IVUS corresponded with hyperplasia of myofibroblast-like cells in abundant myxoid matrix. The hyper-reflective layer by IVUS extended to the outer edge of the vessel and corresponded to a less myxoid area with more collagen, smooth muscle and elastic fibers. The outer less reflective edge of the IVUS image correlated with a gradual transition into adventitia. Normal veins had a thin wall, correlating with histologically normal cellular and extracellular components, without intimal proliferation. IVUS may provide further understanding of the anatomy and mechanisms of pediatric pulmonary vein obstruction.

## 1. Introduction

Pediatric intraluminal pulmonary vein stenosis (PVS) occurs as a result of hyperplasia (neo-intimal proliferation) of myofibroblast-like cells in a myxocollagenous matrix [1,2,3]. PVS is diagnosed when there is evidence of luminal vein narrowing as seen by computed tomography or cardiac angiography and confirmed by surgical biopsy at the time of pulmonary vein repair [4]. In the absence of a tissue diagnosis, it is sometimes challenging to differentiate between PVS and other causes of pulmonary venous obstruction that also result in similar luminal vein narrowing on angiography. These other mechanisms of obstruction include post-operative anastomosis narrowing following anomalous vein repair, obstructive atrial tissue and other anatomic lesions that result in distortion and/or compression of the pulmonary vein [5,6,7]. These angiographic mimics of PVS are readily treatable, when indicated, using transcatheter and/or surgical techniques. PVS, on the other hand, is associated with a high incidence of restenosis and decreased survival, and it is a target for medical therapy [1,2,4,8,9,10]. This clinical distinction is crucial, since a confirmed diagnosis of PVS has direct implications on the prognosis and management of the disease. While angiography can only characterize the vein lumen, intravascular ultrasound (IVUS) can visualize vein lumen and the wall architecture. It is proven to overcome the limitations of angiography in adult venous pathology [11,12,13,14]. In pediatric patients with known PVS, preliminary in vivo IVUS images demonstrate luminal narrowing and wall thickening [15]. How the IVUS-delineated constituents of wall thickening correlate with histologic findings is currently unknown. We hypothesized that analysis of postmortem and in vivo PVS IVUS findings along with corresponding histologic features would help define the ultrasonographically observed constituents of diseased vein wall.

## 2. Materials and Methods

### 2.1. Postmortem Samples

Formalin-fixed heart/lung specimens from patients with PVS were identified in the Boston Children’s Hospital Cardiac Registry. Control vessels included uninvolved pulmonary veins from two of the PVS patients and a normal pulmonary vein from an infant without PVS (obtained at the time of autopsy). Data collection included patient demographics, intervention history and angiographic findings (if applicable). The organs were submerged in water and intact target veins were imaged with IVUS using the Visions PV.014P RX digital catheter (Volcano Core Mobile System [Phillips Corporation, Amsterdam, The Netherlands]). Pulmonary veins were cut into circumferential sections 1–2 mm thick, beginning at the veno-atrial junction (IVUS location) and extending distally/upstream towards the lung. The pulmonary vein sections were marked with ink to maintain orientation during processing. Paraffin-embedded tissue sections were stained with hematoxylin and eosin (H&E), Miller’s elastic, and Masson trichrome. Antibody to alpha smooth muscle actin (clone ASM-1; Leica, Buffalo Grove, IL, USA) was used for immunohistochemical staining. IVUS images were correlated with histologic findings.

### 2.2. In Vivo Samples

We performed a single-center retrospective review of all patients who underwent cardiac catheterization for PVS and subsequently underwent pulmonary vein surgical repair from 1 November 2017, to 31 December 2019. Study patients included those who underwent IVUS evaluation of their pulmonary veins at the time of cardiac catheterization and subsequently had pulmonary vein biopsy at the time of their surgical repair. Most of these patients were previously reported [15]. Data collection included patient demographics, intervention history and angiographic findings. Pulmonary vein biopsy specimens, which had been procured for clinical purposes and submitted to pathology, were retrieved from departmental archives. Examined material included H&E-stained sections in all cases, and elastic, trichrome, and SMA stains in a subset of cases. The IVUS images were correlated with the histologic findings.

### 2.3. Intravascular Ultrasound in Live Patients

IVUS was performed by first accessing the vein of interest with an 0.014′’ wire and advancing the wire into a distal segment. The monorail Visions PV.014P RX digital catheter was flushed, connected to the Volcano Core Mobile System and then advanced over the wire (via 5fr guide catheter, short sheath or Mullins long sheath) into the pulmonary vein to the origin of a primary segment. The image was optimized by adjusting the gain and the imaging diameter (maximum: 20 mm). A recorded manual pullback was performed from the primary segment to the left atrium and was repeated as necessary.

### 2.4. Measurements

All IVUS images underwent measurements (mm) of vein diameter, vein lumen, and vein wall thickness at the veno-atrial junction. The inner periluminal less reflective layer and outer hyper-reflective layer of the vein wall, when present, were measured. When the vein was non-circular or the wall thickness was variable, an average of the minimum and maximum measurements was used. A default vein lumen of 2 mm was used when the IVUS catheter occluded the vein lumen. Percent wall thickness was defined as (vein diameter–vein lumen)/vein diameter × 100. Percent of wall thickness that is the inner layer was defined as (inner layer thickness/wall thickness) × 100. Histologic measurements were assessed using cellSens imaging software, version 2.3 (Olympus, Center Valley, PA, USA).

### 2.5. Ethics Statement

This study was conducted according to the guidelines of the Declaration of Helsinki and was approved by the Boston Children’s Hospital Institutional Review Board (protocol number: IRB-P00033576, date of approval 11/5/2019 [live patients], IRB-P00010717, date of approval 10/25/2013 [autopsy patient]) and the Postmortem Research Committee (Cardiac Registry specimens; protocol number not applicable). Given the retrospective nature of the study, the requirement for written consent was waived by the Institutional Review Board for the live patients. For the postmortem specimens, consent for research was granted by the next of kin.

## 3. Results

### 3.1. Patients

#### 3.1.1. Postmortem Samples

Pulmonary veins from six patients with known PVS were investigated (5 veins with PVS and two veins without PVS). One postmortem pulmonary vein from a patient without PVS was also examined, for a total of eight veins (five veins with PVS and three control veins). For the PVS patients, the median age at death was 16.5 months (range: 5–21) and median postmortem weight was 7 kg (range: 4.2–9.6). Diagnoses were primary PVS (structurally normal heart and no history of prematurity or chronic lung disease) in three and congenital heart disease in one (hypoplastic left heart syndrome/total anomalous pulmonary venous connection [TAPVC]) [16]. The veins with PVS previously underwent a median of five surgical/transcatheter interventions (range: 5–12). All patients had recurrent long-segment pulmonary vein involvement of the affected veins, with stenosis starting at the entrance into the left atrium and extending into segmental branches of the involved pulmonary veins, as evidenced on last available angiography. The control patients included a 5-month-old ex 24-week premature infant with three-vessel PVS (control vein spared), an 8-month-old with truncus arteriosus s/p repair with four-vessel PVS (spared veno-atrial junction in target vessel; distal disease only) and a 7-month-old with long-gap esophageal atresia, tracheoesophageal fistula and multiple other anomalies who died in the setting of an unexplained cardiac arrest (Table 1).

#### 3.1.2. In Vivo Samples

Seven pulmonary veins from four live patients were investigated. The median age at catheterization/IVUS evaluation was 16 months (range: 3–26) and median weight was 8.1 kg (range: 4.7–9.3). Two patients had PVS associated with prematurity/chronic lung disease and two had congenital heart disease (1 double outlet right ventricle/TAPVC, one ventricular septal defect). Five pulmonary veins in three patients had IVUS imaging and clearly labeled biopsy specimens for comparison. One patient had IVUS imaging of the left upper and left lower pulmonary veins and had only one biopsy sample labeled “left pulmonary vein.” Pulmonary vein biopsy at the time of surgical repair was performed at a median of 4.5 months (1, 3, 6, 14 months) following IVUS evaluation. All pulmonary veins developed restenosis after surgery requiring a median of 2 (range: 2–4) transcatheter reinterventions.

### 3.2. Intravascular Ultrasound

#### 3.2.1. Postmortem PVS Veins

**Figure 1:**
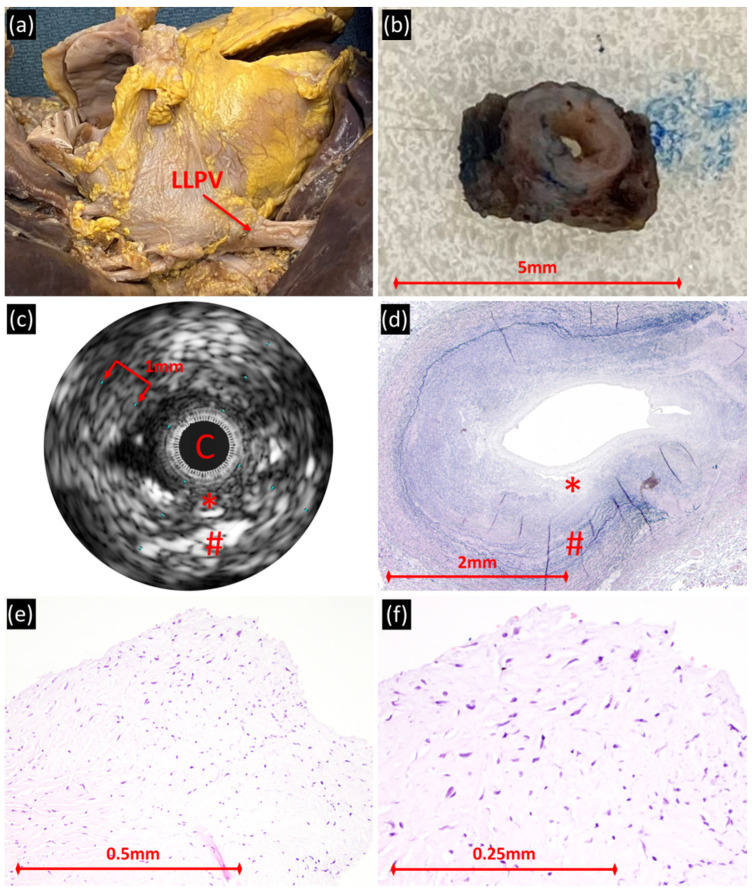
Left lower pulmonary vein of patient with hypoplastic left heart syndrome, total anomalous pulmonary venous connection and pulmonary vein stenosis (s/p surgical ostial enlargement, multiple angioplasties) who died at age 21 months. (**a**) Gross heart-lung specimen (posterior aspect), with imaged/sampled left lower pulmonary vein (LLPV) identified. (**b**) Pulmonary vein section at the veno-atrial junction with grossly thickened wall. (**c**) IVUS demonstrating circumferential wall thickening with up to two layers of variable echogenicity. C = IVUS catheter. (**d**) Histologically, mural architecture includes variable density of elastic fibers and areas of discontinuity/disruption of the layers; the fibroblastic proliferation rich in myxoid matrix predominates superficially/subendothelially (Miller’s elastic; original magnification, 40×). The lower reflective periluminal layer by IVUS corresponds with hyperplasia of myofibroblast-like cells in abundant myxoid matrix [*]. The hyper-reflective layer by IVUS extends to the outer edge of the vessel and corresponds to a less myxoid layer with more collagen, smooth muscle and elastic fibers [#]. The outer less reflective edge of the IVUS image correlates with a gradual transition into adventitia. (**e**,**f**) Intimal myofibroblastic proliferation with amphophilic myxoid extracellular matrix (hematoxylin and eosin; original magnifications, 200× and 400×).

**Figure 2:**
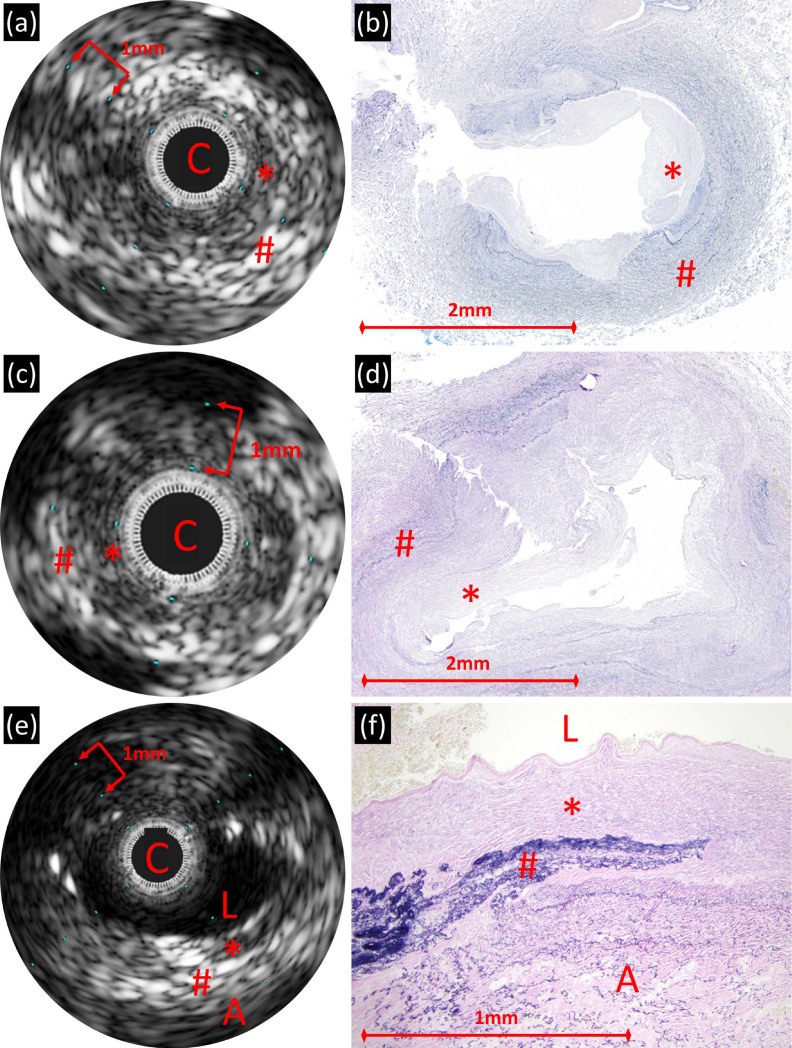
Pulmonary veins from three separate patients with primary pulmonary vein stenosis, dying at ages 5 months ((**a**,**b**); right lower pulmonary vein), 12 months ((**c**,**d**); left lower pulmonary vein), and 19 months ((**e**,**f**); right upper pulmonary vein). In all patients, IVUS demonstrates mural thickening with up to two layers of variable echogenicity ((**a**,**c**,**e**); C = IVUS catheter, L = lumen, A = adventitia). (**b**,**d**) Histologic sections confirm the mural thickening, with variable density of elastic fibers and (**f**) areas of disruption/discontinuity of the elastic layer (Miller’s elastic; original magnification, 40×, 40×, and 100×, respectively). In all cases, the lower reflective periluminal layer by IVUS correlates with the elastic-poor regions of fibromyxoid proliferation (*); higher reflective areas correspond to elastic-rich regions of the vessel wall (#).

In the diseased veins, IVUS demonstrated wall thickening with up to two layers of variable echogenicity, often with indistinct borders (Figure 1 and Figure 2). The lumen, when not occluded by the IVUS catheter, was non-reflective and ovoid in shape. The inner, periluminal layer encompassed the majority of the vessel wall. This less reflective layer was predominantly circumferential (one sample had eccentric pattern), but with variability to its thickness. The outer layer was hyper-reflective and either had a clean, nearly circumferential border or had thicker, interrupted patches. The outer edge of the vessel was marked by an abrupt change to a non-reflective signal, a gradual fading of signal or a change in reflection caused by adjacent anatomy. The mean vein diameter, absolute wall thickness, percent wall thickness and percent of wall thickness due to the inner layer was 5 ± 1.2 mm, 1.4 ± 0.5 mm, 52 ± 6%, and 60 ± 10%, respectively (Table 2).

#### 3.2.2. In Vivo PVS Veins

**Figure 3:**
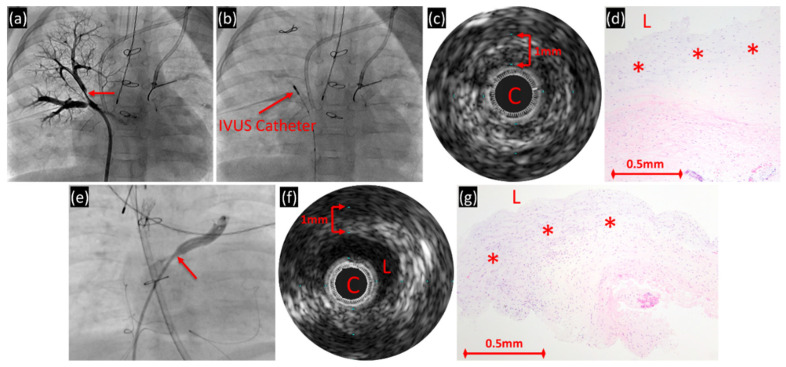
(**a**–**d**) Right upper pulmonary vein of 26-month-old with ventricular septal defect and pulmonary vein stenosis. (**a**) Angiography of the right upper pulmonary vein demonstrating severe stenosis of the apical segment (arrow) just distal to the stent at the veno-atrial junction. (**b**) IVUS catheter (arrow) at the level of the stenosis. (**c**) IVUS demonstrating circumferential wall thickening with two layers of variable echogenicity with the catheter encompassing the entire vein lumen. C = IVUS catheter. (**d**) Pulmonary vein fragment biopsy sample demonstrating intimal fibromyxoid proliferation (*; hematoxylin and eosin; original magnification, 100×). L = lumen. (**e**–**g**) Left upper pulmonary vein of 3-month-old with double outlet right ventricle, total anomalous pulmonary venous connection and pulmonary vein stenosis. (**e**) Angiography of left upper pulmonary vein with moderate ostial stenosis (arrow). (**f**) IVUS with circumferential wall thickening. (**g**) Pulmonary vein biopsy sample demonstrating intimal fibromyxoid proliferation (*; hematoxylin and eosin; original magnification, 100×).

In the living patients with PVS, IVUS showed similar findings as the diseased postmortem veins in regard to the presence of wall thickening and layers of variable echogenicity (Figure 3). There was less wall thickening (40 ± 6%) as compared to the postmortem samples and most of the wall was inner layer (86 ± 14%) (Table 2). The lumen was visualized in all except one vein (occluded by IVUS catheter) and were either circular or elliptical in shape. No surrounding structures were easily identified.

#### 3.2.3. Control Veins

**Figure 4:**
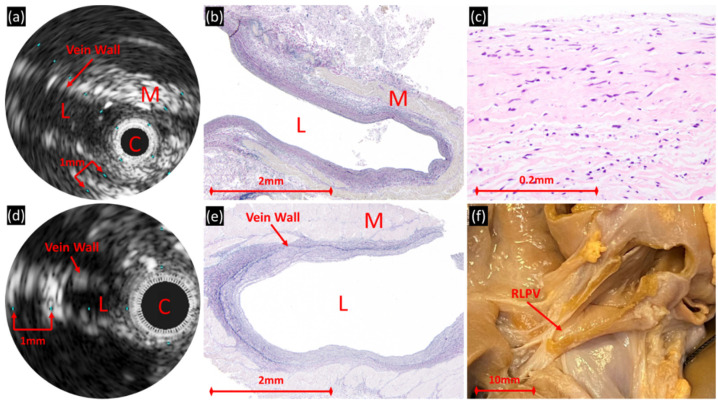
(**a**–**c**) Normal pulmonary vein of two separate patients, one who died at age 7 months in the setting of multiple congenital anomalies including esophageal atresia and a structurally normal heart (right upper pulmonary vein) and (**d**–**f**) one who died at age 8 months in the setting of premature birth and three-vessel pulmonary vein stenosis (right lower pulmonary vein). (**a**,**d**) In both patients, IVUS demonstrates thin-walled veins; there is approximately 0.5 mm between the luminal surface and the hyper-reflective subjacent striated muscle [M] in (**a**). C = IVUS catheter, L = lumen. (**b**,**e**) Histologic examination showed normal wall thickness, delineated the variably thick striated muscle underlying the veins (Miller’s elastic; original magnification, 40×), and (**c**) confirmed the absence of any significant fibromyxoid proliferation (hematoxylin and eosin; original magnification, 400×). (**f**) Gross heart-lung specimen with imaged/sampled right lower pulmonary vein (RLPV) identified.

In normal veins, IVUS showed a single layer wall fairly uniform in echogenicity (Figure 4). The absolute (0.5 ± 0.05 mm) and percent wall thickness (25 ± 4%) was less than the diseased veins (Table 2). The outer edge of the wall is only apparent when there is an abrupt change to non-reflective signal and not obscured by surrounding hyper-reflective tissue.

### 3.3. Histology

#### 3.3.1. PVS Veins

Microscopically, the diseased veins showed fibroblastic proliferation with areas rich in myxoid matrix as well as areas with abundant collagen and elastic fibers. The autopsy samples afforded a comprehensive assessment of the vessel architecture, as well as assessment of the relationship to underlying tissue. Vessels were circumferentially affected by the proliferation, generally concentrically but with some variation in thickness within each circumferential section examined. In general, the most superficial layers of diseased vessels had the greatest amount of myxoid matrix and showed scant small elastic fibers. In a subset of cases the subepithelial myxoid proliferation was devoid of elastic fibers. Smooth muscle actin highlighted myoid cells throughout the vessel wall, typically most concentrated immediately subendothelially. The smooth muscle-rich portion of the vein ranged from 340–1000 µm in thickness. Discrete vein layers were obscured by scarring, including collagen deposition and elastic degeneration. A subjacent variably continuous striated myocyte (“myocardial”) layer was observed in the more proximal sections. The adventitia consisted of loosely arranged elastic-rich fibroconnective tissue containing blood vessels and peripheral nerves. Elastic tissue was prominent around myocardial bundles. Fibroinflammatory response to foreign material was observed focally within adventitia. Of note, some of the ink marking the orientation of the postmortem pulmonary vein specimens was lost during processing in a subset of samples. Other orienting marks included longitudinal cuts through veins previously opened at autopsy, which maintained reasonable orientation in these samples. The biopsies sampled from living patients showed fibroblastic proliferation, but architectural information was comparatively limited; it was often difficult to orient superficial from deep.

#### 3.3.2. Control Veins

Normal veins demonstrated a thin vessel wall. There was scant myxoid matrix observed, present focally immediately subjacent to the endothelium. Elastic fibers were numerous in the wall and in the surrounding adventitia. In the planes examined, they appeared discontinuous. The distinction between vessel and subjacent connective tissue was indistinct. Variable amounts of cardiac muscle were observed. Smooth muscle actin highlighted intramural small myoid cells, concentrated immediately subjacent to the endothelium. The smooth muscle-rich portion of the vessels measured approximately 230 µm in thickness. The distance from the luminal surface to the subjacent cardiac muscle was consistently 260–270 µm.

### 3.4. IVUS/Histology Correlation

#### 3.4.1. Postmortem PVS Veins

**Figure 5:**
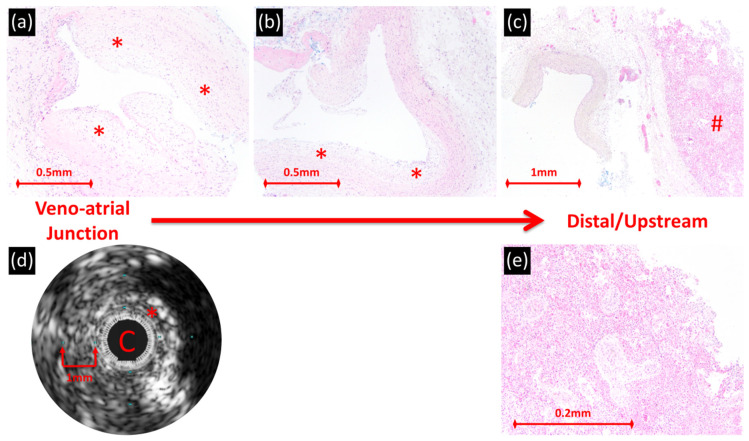
Right upper pulmonary vein of patient with primary PVS who died at age 5 months. (**a**–**c**) Serial cross sections of the vessel progressing from near the veno-atrial junction to the lung, show circumferential fibromyxoid proliferation ((**a**); original magnification, 100×), transitioning more distally/upstream to eccentric fibromyxoid proliferation [*] ((**b**); original magnification, 100×) followed by normal cellular composition ((**c**); original magnification, 40×) at the connection to lung [#]. (**d**) IVUS at the veno-atrial junction demonstrating occlusion of lumen with the IVUS catheter, circumferential wall thickening with two layers of variable echogenicity; the inner periluminal layer correlates with fibromyxoid proliferation [*]. C = IVUS catheter. (**e**) Lung showing features of chronic venous obstruction including pulmonary arterial hypertensive remodeling and pulmonary capillary hemangiomatosis-like areas. ((**a**–**c**,**e**) all hematoxylin and eosin.).

In diseased veins, the inner lower reflective periluminal layer by IVUS corresponded with hyperplasia of myofibroblast-like cells in abundant myxoid matrix (Figure 1 and Figure 2). The hyper-reflective layer by IVUS corresponded to a less myxoid layer with more collagen, smooth muscle and elastic fibers. The outermost edge of the IVUS image correlated with a gradual transition into adventitia. Veins with higher percent wall thickening, which was predominantly due to the inner less reflective layer, had more extensive fibromyxoid proliferation. Several sections from one vein beginning at the veno-atrial junction and progressing distally/upstream towards the lung were evaluated (Figure 5). In the proximal samples, IVUS and histology demonstrated similar findings as described. The more distal sections demonstrated less fibromyxoid proliferation with a clear edge of transition to normal cellular composition. The distal sections were not imaged by IVUS.

#### 3.4.2. In Vivo PVS Veins

For the in vivo cohort, the inner lower reflective periluminal layer by IVUS corresponded with myofibroblastic/myxoid proliferation. Further histologic information was limited, since anatomic landmarks and detailed architectural features including possible post-intervention scarring were compromised in the setting of small incisional biopsies. Of note, all of the patients had PVS intervention before IVUS and are expected to have had secondary “scarring” changes as a component of the pathology. Correlation with IVUS findings in this group emphasized the dramatically greater anatomic range of sampling and better orientation enabled by IVUS.

#### 3.4.3. Control Veins

In normal veins, the single-layer, uniformly echogenic wall correlated with histologically normal cellular and extracellular components with scant focal fibromyxoid proliferation (Figure 4). The surrounding hyper-reflective areas corresponded to striated muscle.

## 4. Discussion

Our study identifies the histologic correlates of the intravascular ultrasound findings in pediatric patients with intraluminal pulmonary vein stenosis. In both our ex vivo and in vivo models, IVUS demonstrated pulmonary vein wall thickening with additional echogenic layers as compared to controls. In particular, diseased veins had an additional periluminal layer which correlated with the histologic findings of fibroblastic proliferation in a prominent myxoid matrix. This confirmed that the wall thickening previously labeled “presumed intimal thickening” in the IVUS assessment of pulmonary vein obstructions is in fact the neo-intimal proliferation that defines PVS [15]. Furthermore, IVUS visualized and quantified the wall thickness of three normal pulmonary veins in children less than one year of age, establishing norms in this age group that can be applied to future research and diagnostics. Lastly, in living patients, IVUS is superior to incisional biopsy in providing better anatomic orientation, circumferential evaluation, and greater longitudinal extent of sampling.

Historically, the diagnosis and severity of PVS has been determined based on the pulmonary vein luminal appearance using computed tomography, magnetic resonance imaging or cardiac angiography [4,10]. Pulmonary vein biopsy can be performed at the time of surgical repair in order to confirm the existence of hyperplasia of myofibroblast-like cells in a myxocollagenous matrix [3]. The limitation of these techniques is angiography’s inability to see the vein wall architecture or other obstructive non-opacified structures and not all patients undergo surgery and pulmonary vein biopsy for confirmation. Our initial experience in the use of IVUS in evaluating pediatric pulmonary vein obstructions demonstrated that IVUS can delineate ostial narrowing secondary to wall thickening, ostial narrowing without wall thickening and pulmonary vein distortion or compression [15]. It was noteworthy in this study that the veins with wall thickening were more likely to require reinterventions. All diseased veins in our study required multiple reinterventions either leading up to death in the postmortem samples or following surgical pulmonary vein repair in the in vivo samples. Thus, IVUS may be able to define pediatric pulmonary vein wall characteristics that are associated with future restenosis.

By characterizing the IVUS findings in patients with histologically confirmed PVS, this study may assist operators in ruling in or ruling out PVS in pulmonary veins with angiographic narrowing. This distinction has important prognostic and management implications. IVUS may be particularly helpful in determining the presence of individual vein PVS when there is an additional mechanism of obstruction such as extrinsic anatomic distortion/compression and equivocal angiography (commonly as left lower vein courses between the aorta and a heart mass) or in repaired total anomalous veins with a confluence obstruction. For example, patients with repaired total anomalous veins can develop a surgical anastomosis confluence obstruction with or without development of PVS in the individual pulmonary veins, the former being associated with a worse outcome [5]. IVUS may be helpful in delineating the status of the individual veins in these patients and determine who may be higher risk. Further study is required to determine if the presence and severity of wall thickening (particularly the inner periluminal layer) by IVUS correlates with prognosis.

One of the postmortem PVS veins demonstrated circumferential fibromyxoid proliferation which extended distally/upstream and finally transitioned to normal cellular composition in the intrapulmonary vein. Disease extending distally is the presumed mechanism when angiography demonstrates diffuse luminal narrowing and is a poor prognostic sign [10]. Further, fibromyxoid proliferation extending to intrapulmonary pulmonary veins is apparent by lung biopsy in a subset of patients with PVS [17]. Anecdotally, some pulmonary veins with diffuse luminal narrowing are veins with severe ostial stenosis plus flow redistribution to unobstructed veins and are salvageable once the proximal obstruction is relieved. Whether or not IVUS can differentiate between these two mechanisms and whether the length of wall thickening as seen in IVUS is a surrogate of disease severity and vein/patient prognosis is a focus of current investigation by our group.

## 5. Limitations

The small sample size was a potential weakness of the study. However, PVS is a rare disorder, and the availability of retained postmortem samples from research-consented autopsies is, of course, limited. All of the studied patients had histories of previous therapeutic interventions which could have resulted in superimposed vessel scarring, a process that may be intrinsically different from PVS without previous intervention, as might be encountered at initial diagnosis. The mural disruption, fibroinflammatory response to foreign material, areas of dense collagen deposition, and a component of the myofibroblastic proliferation, for example, may be related to previous surgery. Although our study was not designed to establish which components of disease were primary versus secondary, the data notably showed that IVUS was able to delineate many of the disease features, whether primary or influenced by previous procedures. Finally, our study did not include patients with other causes of narrowed pulmonary veins besides PVS (distortion/compression, surgical scar, obstructive atrial tissue, ostial narrowing without wall thickening [15], in stent restenosis, iatrogenic), since we lacked patients and pathology material for study from these types of patients.

## 6. Conclusions

Our study identified the histologic correlate for intravascular ultrasonographic findings in the setting of intraluminal PVS using both ex vivo and in vivo models. IVUS may provide further understanding of the anatomy and mechanisms of pediatric pulmonary vein obstruction.

## Figures and Tables

**Table 1:** Patient Demographics.

	Median (Range) or *n* (%)		
Patient Demographics	Postmortem PVS Patients (*n* = 4)	In Vivo Patients (*n* = 4)	Control Patients (*n* = 3)
Age at Death or IVUS Catheterization (months)	16.5 (5–21)	16 (3–26)	7.5 (7–8.4)
Postmortem or IVUS Catheterization Weight (kg)	7 (4.2–9.6)	8.1 (4.7–9.3)	4.9 (3.9–7)
Pulmonary Vein Imaged	5	7	3
Right Upper PV	2 (40)	2 (29)	2 (67)
Right Lower PV	1 (20)	1 (14)	1 (33)
Left Upper PV	0 (0)	3 (43)	0 (0)
Left Lower PV	2 (40)	1 (14)	0 (0)
Pulmonary Vein Diagnosis			
Primary	3 (75)	0 (0)	0 (0)
Prematurity	0 (0)	2 (50)	1 (33) ^1^
Congenital Heart Disease	1 (25)	2 (50)	1 (33) ^2^
Other Diagnosis			1 (33) ^3^

IVUS = intravascular ultrasound, PV = pulmonary vein. ^1^ Ex 24-week premature infant, chronic lung disease, three-vessel pulmonary vein stenosis (imaged vein spared). ^2^ Truncus arteriosus s/p repair, four-vessel pulmonary vein stenosis with sparing of veno-atrial junction in target vein. ^3^ Ex 33-week premature infant, h/o esophageal atresia s/p repair, tracheobronchomalacia, s/p aortopexy.

**Table 2:** Intravascular Ultrasound Pulmonary Vein Wall Characteristics.

	Mean ± Standard Deviation or % (Range)		
Vein Characteristics	Postmortem PVS Veins (*n* = 5)	In Vivo PVS Veins (*n* = 5)	Control Veins (*n* = 3)
Vein Diameter (mm)	5.0 ± 1.2	5.1 ± 0.7	4.1 ± 1.1
Lumen Diameter (mm)	2.4 ± 0.8	3.1 ± 0.7	3.0 ± 0.8
Wall Thickness (mm)	1.4 ± 0.5	1.0 ± 0.2	0.5 ± 0.05
Inner Layer Thickness (mm)	0.8 ± 0.2	0.9 ± 0.3	n/a
Outer Layer Thickness (mm)	0.6 ± 0.6	0.5 ± 0.2	n/a
Percent Wall Thickness (%) ^1^	52 (37–69)	40 (32–50)	25 (20–27)
Percent of Wall Thickness that is the Inner Layer (%) ^2^	60 (52–79)	86 (69–100)	n/a

^1^ ((Vein Diameter–Lumen Diameter)/Vein Diameter) × 100. ^2^ (Inner Layer Thickness/Wall Thickness) × 100. When the vein was non-circular or the wall thickness was variable, an average of the minimum and maximum measurements was used. A default vein lumen of 2 mm was used when the IVUS catheter occluded the vein lumen.

## Data Availability

The data presented in this study are available on request from the corresponding author. The data are not publicly available in order to maintain patient privacy.
